# Are there decision support tools that might strengthen the health system for perinatal care in South African district hospitals? A review of the literature

**DOI:** 10.1186/s12913-019-4583-2

**Published:** 2019-10-22

**Authors:** Ntombifikile Maureen Nkwanyana, Anna Silvia Voce

**Affiliations:** Discipline of Public Health Medicine, College of Health Sciences, University of KwaZulu-Natal, George Campbell Building Room 215, Howard Campus, Durban, KwaZulu-Natal Province South Africa

**Keywords:** Decision support tool, Health system performance, Perinatal care, District hospital

## Abstract

**Background:**

South Africa has a high burden of perinatal deaths in spite of the availability of evidence-based interventions. The majority of preventable perinatal deaths occur in district hospitals and are mainly related to the functioning of the health system. Particularly, leadership in district hospitals needs to be strengthened in order to decrease the burden of perinatal mortality. Decision-making is a key function of leaders, however leaders in district hospitals are not supported to make evidence-based decisions. The aim of this research was to identify health system decision support tools that can be applied at district hospital level to strengthen decision-making in the health system for perinatal care in South Africa.

**Methods:**

A structured approach, the systematic quantitative literature review method, was conducted to find published articles that reported on decision support tools to strengthen decision-making in a health system for perinatal, maternal, neonatal and child health. Articles published in English between 2003 and 2017 were sought through the following search engines: Google Scholar, EBSCOhost and Science Direct. Furthermore, the electronic databases searched were: Academic Search Complete, Health Source – Consumer Edition, Health Source – Nursing/Academic Edition and MEDLINE.

**Results:**

The search yielded 6366 articles of which 43 met the inclusion criteria for review. Four decision support tools identified in the articles that met the inclusion criteria were the Lives Saved Tool, Maternal and Neonatal Directed Assessment of Technology model, OneHealth Tool, and Discrete Event Simulation. The analysis reflected that none of the identified decision support tools could be adopted at district hospital level to strengthen decision-making in the health system for perinatal care in South Africa.

**Conclusion:**

There is a need to either adapt an existing decision support tool or to develop a tool that will support decision-making at district hospital level towards strengthening the health system for perinatal care in South Africa.

## Background

The high rate of perinatal mortality is a global public health concern. Worldwide, there are approximately 4.6 million perinatal deaths every year with more than 1 million stillbirths occurring during the intrapartum period [1, 2]. The majority of deaths occur in low- and middle-income countries (LMIC), with the rural poor most at risk [2–4]. Perinatal mortality imposes huge economic and psychosocial consequences on mothers and families [5, 6]. Moreover, perinatal deaths have undesirable psychological effects on health care providers [5, 7, 8]. The current state of perinatal outcomes calls for urgent implementation of evidence-based interventions in order to reduce the burden of perinatal deaths together with the associated adverse effects.

South Africa is a middle-income country with a high burden of perinatal deaths in spite of the availability of evidence-based interventions [9–12]. Specifically, out of 1000 births in the public sector, approximately 33 babies are born dead or die within the first 7 days of life, with most deaths being preventable [10]. Apart from the medical complications, such as spontaneous preterm labor and intrapartum asphyxia, health system administrative factors as well as health care provider-related problems are major contributors to perinatal deaths in South Africa [10, 12, 13]. Specifically, administrative factors contributing to perinatal mortality include inadequate equipment to provide optimal perinatal care, lack of transport and inadequate theatre facilities [14, 15]. Similarly, provider-related factors include failure to detect fetal distress, delay in referring patient for secondary treatment and delay in calling for expert advice [14, 15]. Therefore, there is a need to strengthen the health system for perinatal care in South Africa in order to realize the potential impact of existing lifesaving interventions on perinatal outcomes [9, 11].

A majority of preventable perinatal deaths in the public sector in South Africa occur in district hospitals [9, 14]. In addition, preventable perinatal deaths that occur in regional hospitals are often due to mismanagement around time of birth in district hospitals [9]. Most preventable perinatal deaths are associated with poor quality of care within poorly functioning health systems [9, 14]. District hospitals do not always provide optimum maternity care and have challenges of staff incompetence as well as a shortage of essential equipment to provide safe perinatal care [14, 16]. Therefore, the health system for perinatal care in district hospitals must be strengthened, as the site for intrapartum care for the majority of mothers who deliver in the public sector in South Africa [10, 11]. Essentially, focusing health system strengthening strategies on leadership in district hospitals should alleviate the burden of perinatal deaths in district hospitals, towards reducing perinatal deaths in South Africa [9, 17].

Setting priorities and allocating resources in a health system is a fundamental function of leadership. However, decisions pertaining to prioritizing areas of intervention in a health system towards improving health outcomes, taken by health facility managers in South Africa are rarely evidence-based [18, 19]. Decision support tools, which are usually computer based information systems, have been developed to support decision processes in various organizations including the healthcare industry, and some have been found useful in prioritizing areas of intervention in health facilities in high-income countries. However, there is currently no identified decision support tool to assist hospital management teams to maximize the effectiveness of their decision processes in setting priorities and allocating resources. Hence, this study aimed to identify health system decision support tools that can be used at district hospital level to strengthen decision-making in the health system for perinatal care in South Africa.

## Methods

### Aim

The aim of the literature review was to identify tools that could support decision-making pertaining to prioritizing areas of intervention towards strengthening the health system for perinatal care in South Africa at district hospital level.

### Design

A structured approach, the systematic quantitative literature review method [20, 21], was conducted to find published articles on decision support tools that have been used to facilitate decision-making in health systems pertaining to perinatal, maternal, neonatal and child health.

### Methods

Articles published in English were sought through the following search engines: Google Scholar, EBSCOhost and Science Direct. The electronic databases searched in EBSCOhost were “Academic Search Complete”, “Health Source – Consumer Edition”, “Health Source – Nursing/Academic Edition’ and “MEDLINE with Full Text”. Journals searched in Science Direct included “Midwifery”, “Public Health and Health Policy”, “Obstetrics, Gynecology and Women” and “Perinatology, Pediatrics and Child Health”. The following search terms were used to retrieve articles, “decision support tool - health system”. In addition, the names of decision support tools identified from articles which met the inclusion criteria were also used as search terms. To ensure optimal coverage, additional articles were found within reference sections of retrieved articles.

### Eligibility criteria

#### Inclusion criteria

Articles meeting the following criteria were included in the review:

1. Original research published in peer reviewed journals
2. Articles reporting on a decision support tool that was applicable to perinatal, maternal, child and neonatal health
3. Articles published in English from 2003 to 2017

#### Exclusion criteria

1. Articles reporting on decision support tools that were not applicable to perinatal, maternal, child and neonatal health
2. Articles reporting on clinical decision making algorithms

### Inclusion of online videos

Online videos published by reputable organizations which detailed a description, development and or the application of identified decision support tools were included in the review.

### Assessment of publications

**Fig. 1:**
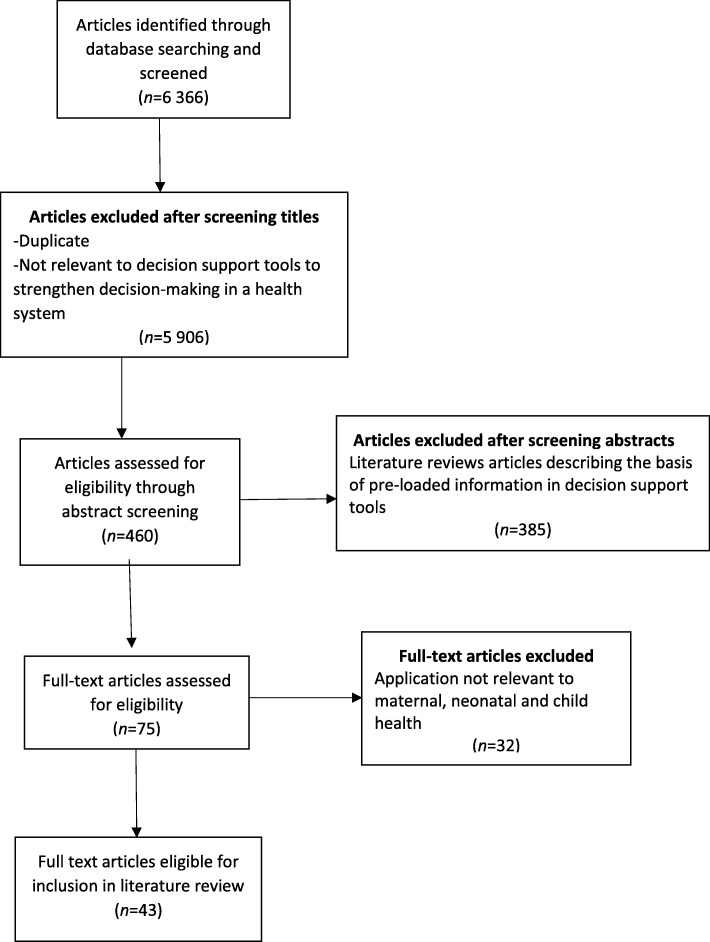
Flow chart mapping out the number of articles identified, screened, excluded together with reasons for exclusion

Overall, the search yielded 6366 peer reviewed articles of which 5906 were excluded after screening article titles as they were either duplicates or not related to decision support tools to strengthen decision-making in a health system. Four hundred and sixty abstracts were screened to assess eligibility for inclusion in the review of which a majority were identified as literature review articles that described the basis of information on mortality estimates, risk factors, demography, intervention coverage as well as impact of several interventions on maternal, neonatal and child health, which has been pre-loaded in decision support tools. In total, the 385 articles which reported on the basis of pre-loaded information were excluded. Seventy-five full text articles were assessed for eligibility and 32 of these were not related to strengthening decision-making in a health system for maternal, neonatal and child health. Ultimately, 43 articles were eligible for inclusion in the literature review. The process of assessment of publications is presented in Fig. 1.

### Constructing the database

A database of articles identified for the review was assembled. For each decision support tool identified, corresponding extracted articles were explored to recognize the following information: 1) the purpose of the decision support tool; 2) preloaded information in the decision support tool; 3) input required from a user prior to implementing the tool; 4) output generated by the tool; 5) assumptions, strengths and limitations of a tool; 6) socio-economic settings where the tool has been applied; and 7) the level of application in the health system. These features are described, for each decision support tool, in the Results section and are summarized in a tabular format.

## Results

Forty-three articles which met the inclusion criteria for review detailed a description of the development of the tool and or the application of the tool. In total, four decision support tools were identified in the articles that met the inclusion criteria. These were: the Lives Saved Tool (LiST), Maternal and Neonatal Directed Assessment of Technology (MANDATE), OneHealth Tool (OHT) and Discrete Event Simulation (DES) [22–25].

Table 1 presents a list of peer-reviewed literature that describes the development of the reviewed decision support tools. Table 2 presents a list of peer-reviewed literature in which the application of identified decision support tools have been reported. Online videos describing development and or application of the tools, derived from reputable websites, are listed in Table 3. The features for each decision support tool are summarized in Table 4.

**Table 1:** Author, year of publication and study location of reviewed journal articles that detail the description of the reviewed decision support tools for maternal and newborn healthcare

Authors	Year of publication	Study Location
Lives Saved Tool		
Jones et al	2003	United States of America
Boschi-Pinto et al	2010	United States of America
Winfrey et al	2011	United States of America
Fox et al	2011	United States of America
^b^Walker et al	2013	United Sates of America
^a^Bollinger et al	2017	Unites States of America
Maternal and Neonatal Directed Assessment of Technology model		
McClure et al	2013	Sub-Saharan Africa
^b^Walker et al	2013	United Sates of America
OneHealth Tool		
^b^Walker et al	2013	United Sates of America
^a^Bollinger et al	2017	Unites States of America
Discrete Event Simulation		
Allen and Wigglesworth	2009	United Kingdom
Goldsman et al	2009	United State of America
Hamrock et al	2013	United State of America

^a^Derived information for LiST and OneHealth Tool

^b^Derived information for LiST, MANDATE and OneHealth Tool

**Table 2:** Author, year of publication and study location of reviewed journal articles that detail application of the reviewed decision support tools for maternal and newborn healthcare

Authors	Year of publication	Study Location
Lives Saved Tool		
Chopra et al	2009	South Africa
Hazel et al	2010	West Africa
Bryce et al	2010	Burkina Faso, Ghana and Malawi
Friberg et al	2010	Sub-Saharan Africa
Acuin et al	2011	Southeast Asia
Pattinson et al	2011	International
Amouzou et al	2012	Niger
Walker et al	2013	Sub-Saharan Africa
Jo et al.	2014	Bangladesh and Uganda
Johri et al	2014	Burkina Faso
Homer et al	2014	International
McPake et al	2015	Ethiopia, Indonesia and Kenya
Michalow et al	2015	South Africa
Chola et al	2015	South Africa
McGee et al	2016	South Africa
Keita et al	2017	Mali
Maternal and Neonatal Directed Assessment of Technology model		
Goldenberg et al	2014	Sub-Saharan Africa
Kamath-Rayne et al.	2015	Sub-Saharan Africa and India
McClure et al	2015	Sub-Saharan Africa
Harrison et al	2016	Sub-Saharan Africa
Griffin et al	2017	Sub-Saharan Africa
Herrick et al	2017	Sub-Saharan Africa
OneHealth Tool		
Adesina and Bollinger	2013	Five African Countries
Stenberg et al	2014	International
Boyle et al	2015	International
Stenberg et al	2017	International
Keen et al	2017	Sierra Leone
Discrete Event Simulation		
Cochran and Bharti	2006	United States of America
Jacobson et al	2006	United States of America
Oh and Chow	2011	Singapore
Zhu Z	2011	Singapore
Griffin et al	2012	Atlanta
Mielczarek and Uzialko-Mydlikowska	2012	Poland

**Table 3:** Online videos detailing development and application of the identified decision support tools maternal and newborn healthcare

Decision Support Tool	Author	Title	website
Lives Saved Tool	Lives Saved Tool	Software demonstration - Subnational Wizard	https://www.youtube.com/watch?v=lTZxCJbK8tE
MANDATE Model	MANDATE	Overview of MANDATE	http://www.mandate4mnh.org/content/media_overview/index.html
	MANDATE	Using the MANDATE Web Model	http://www.mandate4mnh.org/content/media_tutorial/index.html
OneHealth Tool	Harmonization for Health in Africa	Introduction on the “One Health Tool “	https://www.youtube.com/watch?v=l9ix5oZ6ETk
OneHealth Tool	Harmonization for Health in Africa	Tutorial on how to use the OneHealth Tool	https://www.youtube.com/watch?v=t1chFnEH9nI

**Table 4:** Summary of the features of reviewed decision support tools

Tool	Lives Saved Tool	MANDATE model	OneHealth Tool	Discrete Event Simulation
Purpose	Estimate the impact of introducing or increasing coverage of maternal, neonatal and child health interventions on mortality [23, 25–27]	Guide users to prioritize allocation of resources towards interventions that have greatest impact in reducing maternal, fetal and neonatal mortality [23, 24, 28, 29]	Enable users to conduct integrated health system planning and costing for various disease programs [23, 25, 30]	Enable users to assess the efficiency of a healthcare delivery system and to forecast the potential impact of implementing changes in the healthcare delivery system [22, 31]
Preloaded Data	a) List of maternal, neonatal and child health interventions [23, 26, 32] b) Estimated baseline coverage of interventions at national level [23, 26, 27, 33, 34] c) Recent estimate of the effectiveness of interventions that are introduced or scaled up [26, 33] d) Population risk factors and causes of death relating to maternal, neonatal and child health [26, 34, 35]	a) List of main clinical conditions that contribute to maternal, fetal and neonatal mortality [24, 29] b) Clinically proven methods to prevent, diagnose and treat maternal, fetal and neonatal conditions [29] c) Baseline estimates of utilization, penetration and efficacy of interventions at a national or international level [24, 36]	a) Epidemiological and demographic data for various countries [37]	*Not specified*
Required Input	a) Geographical region where interventions will be applied [38] b) Projected coverage of interventions to be assessed [26, 34, 39] c) Measures of maternal, neonatal and child health status at national level [26]	a) Timeframe of assessment [40] b) Geographical region where intervention will be applied [40] c) Intended levels of utilization, penetration and efficacy in different settings, either at home, clinic and or in hospital [40]	a) Geographical region for which integrated planning and costing is conducted [37] b) Current state of the building blocks of the health system [30, 37] c) Coverage targets and disease program costs [30] d) Settings in which interventions will be implemented, whether it is through community based programs, community health centers, hospitals or national level [37, 41]	Current operational state of the health system [22] a) Number of service stations b) Number of health professionals available in each service station c) Medical resources available d) Arrival rates e) Service times
Generated output	a) Estimated number of lives that could be saved by introducing or by increasing coverage of maternal, neonatal and child interventions [9, 35, 42, 43] b) Cost implications for prevented deaths [35, 42, 43]	Estimated number of lives saved by increasing utilization and penetration of maternal, fetal and neonatal interventions [24, 29, 36, 44, 45]	a) Number of health care professionals needed to implement intervention(s) [30, 37] b) Medical resources needed for implementation of interventions [30, 37] c) Expected costs necessary for proper implementation of interventions [25, 30, 37] d) Number of lives that could be saved by implementing interventions [30, 37]	Performance measures specified by the user such as patient throughput, timeliness of care and resource utilization [22]
Assumptions	a) Mortality rates and causes of death would not change considerably from the baseline estimates [23] b) Estimated impact of interventions on mortality are solely due to the increase in coverage [23, 46] c) Quality of care is maintained while increasing coverage [47]	Efficacy of interventions is the same in different levels of care (i.e. home, clinic or hospital) [28, 44]	Interventions applied in one or more of the following settings: a) Community b) Outreach c) Clinic d) Hospital [41]	Simulation changes at a discrete time interval [48, 49]
Strengths	a) Provides accurate predictions of neonatal and child mortality in diverse geographical settings [35, 50–52] b) Models the impact of a single or integrated interventions [23, 26, 53] c) Avoids overestimating the impact of interventions by considering multiple potential causes of deaths and risk factors within one group of deaths [23]	a) Evaluates the impact of single and integrated interventions [36] b) Evaluates the impact of different types of interventions (preventative, diagnostic and treatment) [24, 28, 36, 44, 54] c) Assesses the impact of transferring mothers and neonates between different levels of care [24, 28, 36, 44]	a) Enables a consolidated analysis across programs while considering financial capacity of the health system [30] b) Incorporates costing of selected non-health sector factors that may have an impact on health outcomes [37]	a) Shows how processes interact as a whole in the system, providing a macro-level view [22] b) Models several processes that occur simultaneously in a health care system [55] c) Effective in allocating scarce resources while minimizing healthcare delivery costs [31]
Limitations	Assessment of impact of interventions is limited to the predefined age intervals which do not cover the perinatal period exclusively [26]	Output does not distinguish whether neonatal deaths occurred within the first 7 days of life or later, as a result the impact of interventions on perinatal outcomes cannot be measured [40]	Output does not distinguish whether neonatal deaths occurred within the first 7 days of life or later, as a result the impact of interventions on perinatal outcomes cannot be measured [37]	*Not specified*
Settings where tool has been applied	Low- and middle-income countries [9, 33–35, 42, 46, 47, 50–53, 56–58]	Low- and middle-income countries [28, 36, 44, 45, 54, 59]	Low, middle and high income countries [25, 60–63]	High-income countries [31, 48, 49, 55, 64, 65]
Level of application	Country, provincial and district level [9, 33, 34, 42, 46, 47, 50–53, 56–58]	National and international [28, 36, 44, 45, 54, 59]	National [25, 60–63]	Facility level [31, 48, 49, 55, 64, 65]

Studies reporting description and or development of identified decision support tools were mainly conducted in high-income studies (HIC).

The Lives Saved Tool and the MANDATE model have been applied in low- and middle-income countries, whereas the DES has only been applied in high-income countries.

### Lives saved tool

The Lives Saved Tool (LiST) assists users to estimate the impact of introducing or increasing the coverage of maternal, neonatal and child health interventions [23, 25–27] The initial purpose of designing the LiST was to estimate the impact of scaling up community based interventions on under five mortality, and details of its construction were published as part of the Child Survival Series in 2003 [66]. Evaluated interventions included preventative programs such as availability of skilled attendant at birth, supply of measles vaccine, antenatal steroids, nevirapine and replacement feeding. Treatment programs evaluated included supply of vitamin A, antibiotics for pneumonia and newborn resuscitation [66]. Since its initial design, LiST has undergone further advancements, including the incorporation of the evaluation of the impact of facility based interventions aimed at improving maternal and birth outcomes, and at reducing neonatal mortality [23, 26] . In 2008, LiST was incorporated into the SPECTRUM software, hence LiST utilizes data in the DemProj and Aids Impact Model (AIM) to generate the desired output [23]. The DemProj gives estimates of population size based on assumptions of fertility, mortality and migration for a country or a region [23]. The AIM estimates the impact of changes of Human Immunodeficiency Virus incidence, prevention and treatment measures on mortality [23]. Further additions have recently been made to LiST to enhance the accuracy of estimates of the cost implications of increasing coverage of interventions [30, 35].

LiST is preloaded with a list of maternal, neonatal and child health interventions, which are relevant for implementation in LMIC [23, 26, 33]. The details of preloaded interventions include baseline coverage estimates of interventions at national level, together with recent estimates of the effectiveness of interventions [26, 27, 33–35, 46]. Furthermore, information on population risk factors and causes of death relating to maternal, neonatal and child health are preloaded in LiST [26, 34, 35]. A user needs to specify the geographical region where interventions will be applied, intended intervention coverage, measures of population health status, as well as an estimate of the effectiveness of the intervention when scaled up in order to generate an output to guide prioritization of interventions [26, 34, 38, 39, 42, 67, 68]. LiST gives an estimate of the number of lives that could be saved after introducing or scaling up interventions as a measure of the impact of interventions as well as an estimate of cost implications for prevented deaths [9, 35, 42, 43].

For any year from 2000 to 2012, LiST has information on mortality, exposures, risk factors, intervention coverage and demography for 90 LMICs [23] . LiST assumes that mortality rates and causes of death would not change considerably from the baseline estimates and that the estimated impact of interventions on mortality are solely due to increase in coverage [23, 46]. Moreover, the tool assumes that quality of service delivery is maintained while increasing coverage [47]. LiST is precise in predicting estimates of the impact of interventions in diverse geographical settings [35, 50–52]. Furthermore, the tool allows users to enter new or future interventions and to assess the impact of new interventions in conjunction with existing interventions in saving lives [23, 26, 53]. If multiple interventions are evaluated, LiST prevents overestimating the impact of interventions by considering multiple potential causes of deaths and risk factors within one group of deaths [23]. LiST cannot be used to estimate the impact of interventions on perinatal mortality solely, since the assessment of the impact of interventions is limited to the predefined age intervals which do not cover the perinatal period exclusively [26]. LiST has been useful in strategic planning and in identifying interventions that would have highest impact in saving lives and has been applied in LMIC at national, provincial and district levels [9, 47, 50, 56–58, 69].

### Maternal and neonatal directed assessment of technology model

The Maternal and Neonatal Directed Assessment of Technology (MANDATE) model enables users to prioritize the allocation of resources towards interventions that have the greatest impact in reducing maternal, fetal and neonatal deaths in low-resource settings, particularly sub-Saharan Africa and India [23, 24, 28, 29]. The MANDATE model was developed by the Research Triangle Institute, with the initial description of its application and its construction published in 2013 [24, 70]. No literature was found presenting further development of the model since its original construction.

The MANDATE model is preloaded with a list of main clinical conditions that contribute to maternal, fetal and neonatal mortality as well as proven methods to prevent, diagnose and treat maternal, fetal and neonatal conditions [24, 29]. Furthermore, the MANDATE model is preloaded with baseline estimates of accessibility, utilization and effectiveness of interventions in specific regions [24, 36]. All preloaded data were derived from published literature as well as reputable research websites and databases [24, 29, 44]. MANDATE users need to specify the geographic region where intervention will be applied, timeframe of assessment and the intended level of utilization, penetration and efficacy of interventions in order to generate output to guide prioritization of interventions [40]. The tool gives an estimate of the number of maternal, fetal and neonatal lives that could be saved by increasing utilization and penetration of interventions [24, 29, 36, 44, 45].

The MANDATE model assumes that the efficacy of an intervention is the same when applied at home, clinic or in a hospital setting [28, 44]. The tool can be used to evaluate the impact of one intervention or the impact of a set of integrated interventions and can simulate different scenarios of interventions, enabling comparisons of the impact of different interventions before implementation [36, 44, 54]. The MANDATE model can quantify the effect of maternal conditions on neonatal outcomes [36]. Furthermore, the tool is able to evaluate the impact of preventative, diagnostic and therapeutic technologies as applied in different settings, either at home, clinic or hospital, and can measure the impact of transferring mothers and neonates between different levels of care [24, 28, 29, 36, 44, 54]. However, the MANDATE output does not distinguish whether neonatal deaths occurred within first 7 days of life or later, and as a result the impact of interventions on perinatal outcomes cannot be measured [40]. The MANDATE model has been implemented in LMIC countries at national and international levels [24, 28, 36, 44, 54, 59, 70].

### OneHealth tool

OneHealth Tool enables users to conduct integrated health system planning and costing for various disease specific programs [23, 25, 30]. The OneHealth Tool was developed by the United Nations Interagency Working Group on Costing and was first released in 2012 [71]. During its initial release, OneHealth Tool had planning and costing components for the following disease programs: Tuberculosis, Malaria, Immunization, Water and Sanitation, Reproductive Health, Nutrition and Child Health [71]. No further development of the OneHealth Tool regarding planning and costing of programs relating to maternal, neonatal and child health has been published since its original construction.

OneHealth Tool has incorporated pre-existing United Nations epidemiological reference group models including the LiST, AIM and the Fam Plan model [23, 30, 60–62]. Fam Plan estimates the impact of scaling up family planning on fertility [23]. Thus, OneHealth Tool has access to epidemiological and demographic data that is preloaded in the United Nations epidemiological reference group models [37]. OneHealth Tool users are required to specify the geographical region for which integrated planning and costing is conducted, the current state of the building blocks of the health system, and the settings in which interventions will be implemented [37]. OneHealth Tool provides estimates of the number of health care professionals needed to implement interventions, medical resources needed for implementation of interventions, expected costs necessary for proper implementation of interventions, as well as number of lives that could be saved by implementing interventions [30, 37, 41].

OneHealth Tool assumes that interventions are delivered in one or more of the following settings: directly to the community, through an outreach program, in clinics and in hospitals [41]. OneHealth Tool can perform a consolidated analysis across different disease programs while assessing the impact of implementing interventions on the functioning of the health system and also evaluating feasibility of sustaining interventions with regard to available finances [30]. Moreover, OHT incorporates costing of selected non-health sector factors that may have an impact on health outcomes [37]. Although OneHealth Tool is useful in planning for maternal and newborn health programs, it does not measure the impact of interventions on perinatal outcomes as the output does not indicate the period when neonatal deaths occurred [37]. Currently, OneHealth Tool is used globally for planning and costing at a national level [23, 37, 61–63, 71].

### Discrete event simulation

Discrete Event Simulation (DES) is a statistically based tool that is used to assess the efficiency of a healthcare delivery system and to forecast the potential impact of implementing changes in the healthcare delivery system [22, 31]. DES was initially developed as part of the General Simulation Program (GSP) in the mid-40s [72]. The GSP is a general-purpose simulator which was primarily developed to implement simulations in an industrial setting [72, 73]. Since its initial design, DES has been used in diverse settings including the healthcare system [48, 49].

To initiate simulation, a DES user needs to understand and map the structure and the processes involved in healthcare delivery [64]. Essentially, the user needs to specify the current operational state of the health system with regards to number of service stations, number of health professionals available in each service station, medical resources available, time taken in each service station, arrival rates and service times [22]. DES generates performance measures of the healthcare system according to the user’s specifications. The commonly generated performance measures are patient throughput, timeliness of care and resource utilization [22].

The DES assumes that the clients arrive at a health facility in a time-dependent pattern and the state of the health system changes as clients arrive [48, 49]. DES imitates the operation of the real world system and shows how processes interact as a whole in the system, providing a macro-level view [22]. It is able to model several processes that occur simultaneously in a health care system [55]. For instance, it can incorporate interdependent queues that clients may need to follow in a health facility. DES has been found useful in the allocation of scarce resources while minimizing healthcare delivery costs [31]. It has been used effectively for various healthcare delivery needs, including improvement of patient flow, managing bed capacity, scheduling staff, managing patient admission and in the use of laboratories and pharmacies [22, 48]. DES has been utilized in HIC, at health facility level [31, 48, 49, 55, 64, 65].

## Discussion

This literature review was conducted to identify decision support tools that could be applied at district hospital level to strengthen decision-making in the health system for perinatal care in South Africa. Four decision support tools were identified and reviewed, namely the Lives Saved Tool (LisT), Maternal and Neonatal Directed Assessment of Technology (MANDATE), OneHealth Tool and Discrete Event Simulation (DES) [22–25]. Both LiST and the MANDATE model were designed to support health managers in prioritizing interventions for pregnancy related health issues, primarily at national level [23, 24]. For instance, LiST has been utilized to identify a set of interventions that could save more lives of pregnant women and children and also prevent stillbirths [35]. Similarly, the MANDATE model has been used to estimate maternal deaths, surgeries, and cases of severe anemia prevented through the use of uterine balloon tamponade among women with postpartum haemorrhage [70]. OneHealth Tool enables health managers at national level to conduct an integrated health system planning and costing for various disease programs including reproductive health and child health programs [71]. The Discrete Event Simulation was designed to assist managers improve operational issues at a health facility level [22, 48, 49].

Measurement challenges for the perinatal period were noted in estimates generated by the LiST, MANDATE Model and OneHealth Tool. These tools provide estimates of the impact of interventions on neonatal deaths in predefined age intervals [26, 37, 40]. The limitation of the generated outputs is that estimates do not distinguish if deaths occurred in the first 7 days of life or later [26, 37, 40]. Consequently, the impact of interventions on perinatal outcomes cannot be estimated.

None of the identified decision support tools can be adopted at district hospital level to strengthen decision-making in the health system for perinatal care in South Africa. The LiST, MANDATE Model and OneHealth Tool were not designed for implementation at facility level and have limitations in estimating the impact of interventions for the perinatal period [23, 24, 26, 37, 40, 71]. DES is indeed applicable at facility level; however, it was designed to guide managers improve operational issues of a health system and has not been applied to improve health outcomes of a disease-specific program [22, 48, 49]. Thus, the reviewed decision support tools should be enhanced prior to implementation in the health system for perinatal care in district hospitals in South Africa.

The reviewed decision support tools have been successfully implemented in LMIC and in HIC settings in prioritizing interventions for maternal, child and neonatal health issues [24, 28, 35, 48, 57]. The LiST and the MANDATE model were specifically designed for implementation in LMIC and have been successfully implemented in these settings [9, 52]. The OneHealth Tool has been utilized for various disease programs in LMIC and in HIC [25, 30]. The DES, which is applied at facility level, has only been applied to improve the functioning of obstetric units and primary health care facilities in HIC [48, 64]. Thus, through the application of OneHealth Tool and DES in HIC, decision-making by health managers has been supported at higher levels of management and at facility level. Certainly, improved perinatal outcomes in HIC cannot be solely attributed to the availability and implementation of decision support tools in health facilities. However, health systems in LMIC would benefit from the development and implementation of decision support tools that can be applied at health facility level specifically to strengthen perinatal care.

Poor health system functioning is the main contributor to high perinatal mortality in South Africa [10, 11]. Hence, a decision support tool relevant for implementation in district hospitals should incorporate the elements to address the performance of the health system essential for optimal perinatal care. Particularly in district hospitals, the tool should guide facility managers in optimal allocation of limited financial resources and in optimising efficiencies. Hence, the tool needs to provide guidance with regard to health system components that need to be prioritized. For instance, informed guidance is needed to decide whether priority is given to purchasing of medical equipment, buying essential medicines, hiring additional staff, provision of relevant training to staff, or in strengthening health information systems. Overall, integrating health system performance in a decision support tool will ensure that managers get a holistic view of all health system factors that influence perinatal outcomes.

The Negotiated Service Delivery Agreement in South Africa emphasized the need to strengthen all critical building blocks of the health system in order to improve its performance [74]. Moreover, the recommendations made by the National Perinatal Morbidity and Mortality Committee (NaPeMMCo), regarding essential strategies to decrease perinatal deaths in South Africa, focused on strengthening the health system [11]. Remarkably, the majority of recommendations made by NaPeMMCo were intended to be implemented in health facilities [11]. Therefore, it is important that the health system for perinatal care is strengthened at a facility level in order to reduce the burden of perinatal mortality.

The function of managers in health facilities need to be strengthened to ensure proper implementation of available guidelines. Particularly in district hospitals, stronger leadership and greater local accountability is key to improving quality of care [9]. Certainly, the function of managers in district hospitals in South Africa can be strengthened by introducing the use of a relevant and contextualized decision support tool that incorporates the performance of the health system at facility level for optimal perinatal care. However, existing tools could be adapted for implementation in district hospitals. In particular, the OneHealth Tool appears to be the most relevant tool to be considered for adaption for implementation in district hospitals as it is useful in integrating health system planning and costing [23, 25, 30]. Moreover, it incorporates a majority of health system building blocks in planning [30, 37]. Hence, adaptation of OneHealth Tool for use in health facilities could be beneficial.

## Conclusion

The reviewed decision support tools have been useful in supporting decision-making in the health sector. However, none of the reviewed tools could be adopted at district hospital level to strengthen the health system for perinatal care in South Africa. Therefore, there is a need for existing decision support tools to be adapted for implementation at facility level or for research to be conducted to develop a tool that will support decision-making at district hospital level towards strengthening the health system for perinatal care in South Africa. Nevertheless, availability and implementation of a relevant decision support tool will only yield positive perinatal outcomes provided that there are improvements in management, education and in cultural change.

## Data Availability

The reviewed literature analysed during the current review are available from the corresponding author on reasonable request.

## Abbreviations

AIM: Aids Impact Model
DES: Discrete Event Simulation
GSP: General Simulation Program
HIC: High-income countries
LiST: Lives Saved Tool
LMIC: Low- and middle-income countries
MANDATE: Maternal and Neonatal Directed Assessment of Technology
NaPeMMCo: National Perinatal Morbidity and Mortality Committee
